# Nanoplastics in Aquatic Environments: Impacts on Aquatic Species and Interactions with Environmental Factors and Pollutants

**DOI:** 10.3390/toxics10060326

**Published:** 2022-06-15

**Authors:** Rafael Trevisan, Prabha Ranasinghe, Nishad Jayasundara, Richard T. Di Giulio

**Affiliations:** 1 Department of Biochemistry, Federal University of Santa Catarina, Florianópolis 88037-000, Brazil; 2 Nicholas School of the Environment, Duke University, Durham, NC 27708, USA; prabha.ranasinghe@duke.edu (P.R.); nishad.jayasundara@duke.edu (N.J.); richd@duke.edu (R.T.D.G.)

**Keywords:** plastic pollution, plastics, microplastics, nanoplastics, health, water, effects, accumulation, pollutants, carrier effect

## Abstract

Plastic production began in the early 1900s and it has transformed our way of life. Despite the many advantages of plastics, a massive amount of plastic waste is generated each year, threatening the environment and human health. Because of their pervasiveness and potential for health consequences, small plastic residues produced by the breakdown of larger particles have recently received considerable attention. Plastic particles at the nanometer scale (nanoplastics) are more easily absorbed, ingested, or inhaled and translocated to other tissues and organs than larger particles. Nanoplastics can also be transferred through the food web and between generations, have an influence on cellular function and physiology, and increase infections and disease susceptibility. This review will focus on current research on the toxicity of nanoplastics to aquatic species, taking into account their interactive effects with complex environmental mixtures and multiple stressors. It intends to summarize the cellular and molecular effects of nanoplastics on aquatic species; discuss the carrier effect of nanoplastics in the presence of single or complex environmental pollutants, pathogens, and weathering/aging processes; and include environmental stressors, such as temperature, salinity, pH, organic matter, and food availability, as factors influencing nanoplastic toxicity. Microplastics studies were also included in the discussion when the data with NPs were limited. Finally, this review will address knowledge gaps and critical questions in plastics’ ecotoxicity to contribute to future research in the field.

## 1. Introduction

With the introduction of plastic materials in the early 1900s, a new environmental and health hazard emerged. One century later, the production of plastic resins and synthetic fibers surpassed 450 million tons [1], and plastics became significant contributors to solid waste and litter. Between the 1900s and 2050, humans are expected to generate a total of 26 billion tons of plastic waste. This is four times greater than current levels [1]. Unsurprisingly, the United Nations prioritized plastic waste and management as fundamental environmental concerns in its 2030 Agenda for Sustainable Development.

It is estimated that over 60% of all plastics ever created were dumped in landfills or the natural environment, with 8 million tons of plastic litter entering the ocean each year [1]. Solid waste management is linked to approximately 30 diseases, resulting in the premature death of 400,000 to 1 million people in developing countries [2]. The number of species harmed by litter in the maritime environment is of great concern, and it has increased in recent years. Plastic ingestion, entanglement, novel habitats for microbial colonization, biological dispersion, and exposure to plastic co-contaminants, for example, can have a global impact on over 800 coastal and marine species [3]. The recent wave of public concern toward plastic waste caused by the COVID-19 outbreak highlighted the urgent need to address the problem of plastic pollution. In the midst of the pandemic, the consumption of single-use plastics has surged as an alternative for improved cleanliness and health safety. As a result, plastic waste management is currently under strain, with 129 billion facial masks and 7.8 billion surgical gloves composed of plastic polymers consumed globally each month [4]. Although we have recently begun to see some of the consequences of this growth in plastic litter [5], the majority of the environmental and health consequences are unknown.

Plastic pollution is a global concern, with plastic litter being discovered in several aquatic ecosystems in both urbanized and pristine locations [6,7,8]. Their deterioration or breakdown produces microscopic plastic particles with distinct properties that amplify their environmental impact. As a result, the distribution and negative impacts of small plastic debris have received considerable attention in the last decade. This review will concentrate on the possible harmful impacts of one type of small plastic, nanoplastics (NPs), on aquatic life. It will do so while taking into account their interactions with other environmental elements and complex environmental mixtures. Additional studies on microplastics (MPs) are highlighted in underrepresented areas of NPs research to support and improve the discussion of the potential environmental implications of NPs. 

All studies were selected based on a literature review using the PubMed and Web of Science databases. The search words included the terms “plastics”, “nanoplastics”, or “microplastics” in combination with one of the following keywords: “carriers”, “food”, “interaction”, “microorganisms”, “organic matter”, “pH”, “temperature”, “toxicity”, “salinity”, or “vectors”. The search results were individually analyzed and selected for studies that would fit the scope of this review. Although this is not a systematic review of the topic, this process resulted in 158 selected articles, reviews, or book chapters, which are discussed in six sections. The current section (Section 1) is a brief introduction to the topic of plastic pollution and the goals and structure of the current review. Section 2 will introduce the two major forms of small plastics (MPs and NPs), as well as their origins and dispersion in the environment. Section 3 will summarize the toxicity and mechanisms of action of NPs on aquatic species, from the subcellular to the physiological levels. Section 4 will expand on this subject by considering the capacity of NPs to behave as carriers of pollutants (Section 4.1) and microorganisms (Section 4.2) under environmental conditions, as well as how aging and weathering events may interfere with such effects (Section 4.3). Section 5 will examine the effect of various environmental parameters or stressors on NPs’ toxicity, including temperature (Section 5.1), salinity and pH (Section 5.2), organic matter (Section 5.3), and food availability (Section 5.4). Finally, Section 6 will provide some insights into recent developments in the field of NP ecotoxicity, as well as subjects or questions of considerable concern that are currently poorly understood in anticipation of future research studies. 

## 2. Plastics Breakdown and Small Plastic Particles: MPs and NPs as Ubiquitous Particles

The majority of plastics manufactured today are made from nonrenewable petrochemicals sourced from fossil fuels, natural gas, and coal. Once in the environment, abiotic and biotic processes (e.g., exposure to bacteria, sunlight, heat, and abrasion) will degrade these polymers, resulting in shorter polymers, soluble chemical byproducts, additives, and plastic fragments [9]. These particles, known as MPs (≤5 mm) or NPs (≤1 µm) [10], constitute the most abundant solid waste in aquatic environments [11]. MPs and NPs can also be manufactured and added to a wide range of commercial and industrial products, where they end up in the environment due to improper disposal. Polyethylene (PE), polyesters, polyamide, acrylics, polypropylene (PP), and polystyrene (PS) are the most frequent polymer types of small plastics in aquatic environments [12].

MPs and NPs accumulate in aquatic environments as fragments of larger aquatic plastic debris, from the abrasion of fishing gear and paint used in shipping, or from land-based activities (e.g., solid waste, shedding of synthetic fibers, municipal sewage and sludge, tire wear, and surface runoff) [13]. According to studies, MPs have been found in urban coastal habitats, as well as the most isolated regions of the world [14,15,16,17]. Additionally, because small plastics are virtually everywhere, they accumulate and spread throughout the food web. MPs, for example, have been found in the tissues of many living beings [18,19], in food products, and, most recently, in human feces and placentas [20,21]. However, little is known about NPs’ prevalence in the environment or items for human consumption, since their small size makes sampling, identification, and analysis difficult. Two recent studies demonstrated progress in the chemical analysis of NPs in water. Pyrolysis coupled to gas chromatography–mass spectrometry was first used to determine the presence and the chemical composition of NPs in seawater samples from the North Atlantic Subtropical Gyre [22]. Later, thermal-desorption proton-transfer-reaction mass spectrometry was used to quantify the NPs concentrations and characterize their composition in surface water samples from lakes and streams in Siberia (mean 51 µg L^−1^) and Sweden (mean 563 µg L^−1^) [23]. Furthermore, other studies have reported the release of NPs from the fragmentation of larger plastics (see review [24]), implying that NPs will likely emerge over time from the plastic litter polluting our water bodies. Recently, the role of atmospheric transport of NPs (as well as MPs) from coastal and urban areas to the ocean was proposed as an important contributor to the cycle and burden of small plastics in the oceans [23]. As a result, various studies have indicated that NPs have the potential to pollute aquatic environments, which could lead to a negative impact on these ecosystems.

The impact of small plastic contamination on humans, animals, ecosystems, and their services is currently poorly known and possibly underestimated. Once in the environment, these particles can transport pollutants, microorganisms, and pathogens on their surfaces, and their small size facilitates their absorption, ingestion, and transfer to many organs, amplifying their physiological and environmental effects [10,25,26]. Many studies have indicated that the toxicity of MPs is directly linked to their retention in the intestine and the induction of oxidative stress and inflammatory responses [19]. MPs can also increase energy consumption and modify energy metabolism, decrease the filtration, reproduction, and feeding rates, and disrupt neurological and immune system functioning [27,28,29,30]. When compared to MPs and large plastic particles, NPs have distinct properties that exacerbate their toxicity. They have substantially smaller sizes (on the same scale as macromolecules and organelles), faster rates of tissue infiltration, and the ability to cross biological barriers, for example [31,32]. As discussed below, these characteristics are crucial when examining the mechanisms of toxicity and negative impacts of NPs on aquatic organisms.

## 3. Cellular and Molecular Impacts of NPs on Aquatic Species

### 3.1. The Small Size of NPs as a Key Factor in Their Ecotoxicity

**Figure 1:**
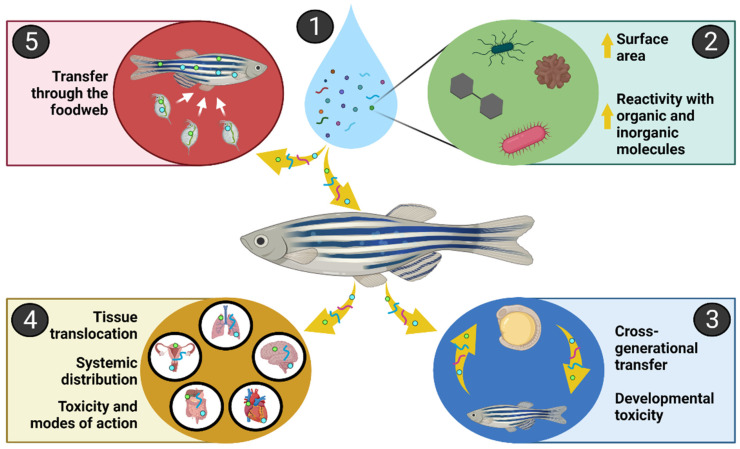
**Fate and impacts of nanoplastics (NPs) in aquatic species.** (**1**) Mixtures of NPs in the aquatic environment; (**2**) interactions of NPs with environmental molecules; (**3**) impacts of NPs on animal development; (**4**) internal distribution and toxicity of NPs; (**5**) transfer and potential biomagnification of NPs through the food web. Please see the text for further clarification. Organs in panel 4 are shown as corresponding to mammalian species for purposes of visualization.

The small size of NPs, like that of other nanoparticles, considerably increases their surface-area-to-volume or mass ratios [33] (Figure 1). This can increase their reactivity with inorganic and organic compounds, including biomolecules and other contaminants [34,35] (Figure 1). It also enhances NP ingestion and absorption by aquatic organisms, as evidenced by the buildup of NPs in various organs, such as the gills, brain, heart, liver, yolk sac, gonads, and digestive organs of vertebrate and invertebrate species [36,37,38], especially at size values below 100 nm (Figure 1). The presence of NPs in several tissues of exposed organisms suggests that their small size increases the likelihood of tissue translocation and systemic distribution, with endocytosis, transcytosis, paracellular diffusion between tight junctions, and uptake through enterocyte barriers all playing important roles [39,40] (Figure 1). Finally, it is crucial to note that studies on NPs smaller than 50 nm suggest that their internalization in endolysosomes, varying retention times, and bioaccumulation in numerous organs (including the gonads) may correlate with a high potential for transfer through the food web and between generations [26,41] (Figure 1). Thus, the presence of plastic materials in aquatic ecosystems, their fragmentation into smaller plastics, and the small size of NPs are all relevant indicators of the potential health concerns regarding organisms exposed to NPs. This is, of course, a summary of the effects of NPs on aquatic species, and parameters such as NP characteristics and the presence of other environmental contaminants or stressors can influence NP behavior, fate, bioaccumulation, and toxicity. We will focus on the interactions of NPs with other environmental conditions and the resulting toxicity to marine and freshwater species in Section 4 and Section 5 of this study. 

### 3.2. Main Biological Effects or Modes of Actions Associated with NPs Toxicity

This review will address the impacts of NPs on aquatic species using terms or categories previously identified as major biological effects of small plastics in fish [42]. This will encourage a more uniform and standardized discussion of the impacts of small plastics on aquatic animals, which will be broadened by the present review to include environmental, multi-stress, or complex mixture settings. The biological effects associated with NPs can be divided into seven major groups or modes of action (MoA): changes in (i) organismal fitness, (ii) circulatory and respiratory systems, (iii) behavioral, sensory, and neuromuscular function, (iv) alimentary and excretory systems, (v) the microbiome, (vi) metabolism, and (vii) immune system. As a result, these categories will be used as a basis in the current review to evaluate the effects of NPs on aquatic species. It should be noted that most of the published research on the ecotoxicity of NPs is based on virgin PS nanoplastics (PS-NPs), and the lack of information on the effects of other frequent polymers, such as PE, PP, polyesters, polyamide, and acrylics particles, remains a significant research gap. 

**Figure 2:**
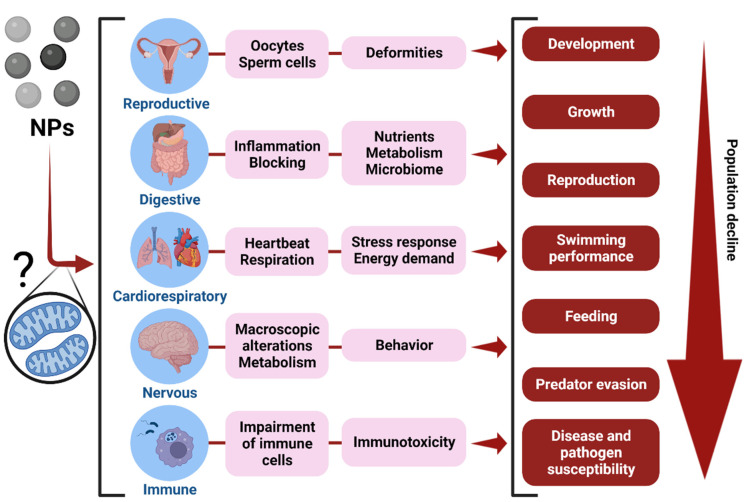
**Potentialcellular and physiological impacts of nanoplastics (NPs) on aquatic organisms.** NPs may accumulate in tissues associated with various physiological systems (blue), prompting intracellular processes (light red) that can influence animal physiology (dark red). Mitochondrial biology and metabolism are commonly affected by NPs, which may mediate some of the major cellular and physiological effects of NPs, although there is a major research gap surrounding this topic. For representative purposes, the figure does not illustrate how specific types or concentrations of NPs will differentially affect the animal at the subcellular, cellular, or physiological levels. Organs are shown as corresponding to mammalian species for purposes of visualization.

NPs accumulate in tissues or organs, such as the gonads, gametes, intestine, liver, pancreas, heart, gills, brain, and immune cells. As a result, they can potentially impact many organs and physiological systems through a broad variety of detrimental molecular and subcellular effects. The impacts of NPs on each tissue or organ will vary, but, in general, they are related to cellular and tissue damage, inflammation, oxidative stress, altered metabolism and neurotransmitter signaling, and organelle disruption. This can result in cellular, physiological, and organismal impacts that may ultimately impact success at the population level (Figure 2). The interaction of NPs with lipid membranes also suggests that they may interfere with the mitochondria (Figure 2), potentially altering the antioxidant system and energy metabolism [43,44,45]. Nevertheless, little is known about the involvement of mitochondrial biology in NP toxicity in aquatic species.

NPs have been reported in bacteria [46], bivalves [47], copepods [43], and fish [26] to raise the levels of reactive oxygen species (ROS), cause the buildup of cellular oxidative damage, and affect the levels, activity, or expression of antioxidant biomolecules (MoA metabolism). Cholinesterase inhibition (MoA: behavioral, sensory, and neuromuscular effect), cytokine production (MoA: immune system), lipid peroxidation (MoA: metabolism), mitochondrial and energy metabolism dysfunction (MoA: metabolism), and lysosomal disruption (MoA: immune, MoA: alimentary and excretory systems) are other subcellular effects commonly associated with NPs exposure [42,45]. In the following sections, we will go through some of these subcellular and molecular changes related to NP toxicity.

### 3.3. NPs as Inducers of Oxidative Stress

As discussed above, exposure to NPs can increase ROS production, modulate the expression or activity of antioxidant defenses, and cause the buildup of oxidative damage. Oxidative stress and lipid peroxidation can activate a number of cellular responses, including the activation of the Nrf2 and MAPKs pathways (e.g., ERK, p38MAPK, and JNK), which can improve the cellular antioxidant capacity in response to pro-oxidative events mediated by NPs [45]. Furthermore, ROS cause organelle malfunction, disruption, or impairment by oxidizing major biological macromolecules and subcellular compartments, such as the nuclei, mitochondria, and lysosomes. Increased ROS generation, for example, can promote lysosomal dysfunction through oxidative damage. Such effects have been linked to increased permeability of the lysosomal membrane, resulting in the leakage of H^+,^ iron, and hydrolytic enzymes that may eventually lead to the loss of cellular function or cell death [48]. The same could occur from the permeabilization of the outer mitochondrial membrane produced by NPs exposure, resulting in the release of cytochrome c and an increase in mitochondrial ROS generation. Aside from the indirect effects of NPs on lysosomes and mitochondria via intracellular ROS, investigations using fish and human models have shown that NPs (PS-NPs, 20 to 200 nm) can interact with or accumulate in these organelles [49,50,51,52], potentially leading to organelle destabilization and enhanced intracellular bioreactivity.

These data imply that oxidative stress is a typical biochemical outcome of NPs exposure. Extracellular ROS generation can occur in response to the photo- and thermal oxidation of NPs, as well as UV radiation, while intracellular ROS production is related to immunological responses (e.g., via NADPH oxidase) or mitochondrial dysfunction, as recently reviewed [45]. Excessive ROS levels can lead to lipid peroxidation, mitochondrial and lysosomal disruption, inflammation, neuronal dysfunction, and the activation of antioxidant responses. These are all important molecular and subcellular mechanisms or targets linked to NPs exposure that are most likely connected to implications at higher levels of biological organization.

### 3.4. NPs as Disruptors of Energy Metabolism

Metabolic shifting is a primary effect of NPs at the molecular or subcellular level (MoA: metabolism). NPs (25 nm PS-NPs) can accumulate in the developing fish pancreas and reduce the serum glucose levels, prompting a cortisol-modulated stress response that leads to enhanced gluconeogenesis and larval swimming activity [53]. Changes in glucose and lipid metabolism, such as reduced glycogen, triglycerides, and total cholesterol, were linked to lower survival rates in freshwater prawns *Macrobrachium nipponense* exposed to NPs (75 nm PS-NPs) [54]. The triglyceride content was likewise reduced in microalgae exposed to NPs (500 nm NH_2_-PS-NPs) [55], indicating that NPs can target both carbohydrate and lipid metabolism across taxa. Recent research suggests that NPs may interfere with mitochondrial metabolism (50 nm NH_2_-PS-NPs) [56] and reduce mitochondrial efficiency in ATP generation (44 nm PS-NPs) [57,58]. Thus, NPs are thought to influence cellular energy metabolism by disrupting the critical metabolic enzymes, hormone levels, and organelle processes involved in energy metabolism. 

To summarize, NP exposure can have a wide range of metabolic consequences, from the hormonal to the metabolic pathway levels. Some of these metabolic changes may be related to the negative effects of NPs on mitochondria, a topic still poorly investigated in aquatic species, especially in invertebrates. Because energy production is a critical determinant of both cellular metabolism and organism physiology and fitness, such impacts can have a variety of consequences for animal growth, development, survival, reproduction, stress tolerance, and pollutant detoxification.

### 3.5. NPs as Modulators of the Nervous and Immune Systems

The inhibition of cholinesterases and the generation of cytokines are described as two significant subcellular actions of NPs [45]. Acetylcholinesterase is an enzyme involved in cholinergic synapses at the neuromuscular junction, and its inhibition may result in neuronal malfunction and behavioral abnormalities (MoA: behavioral, sensory, and neuromuscular function) [37,59]. The capacity of NPs to decrease acetylcholinesterase activity in a wide range of aquatic animals, including polychaeta (100 nm PS-NPs) [60], fish (41 nm PS-NPs) [59], crustaceans (50 nm NH_2_-PS-NPs) [61], and bivalves (110 nm PS-NPs) [62], is of great concern. NPs (100 nm PS-NPs) can also cross the blood–brain barrier, increasing their neurotoxicity potential [63]. Subcellular inflammatory responses to NPs (25–500 nm PS-NPs) include the release of pro-inflammatory and chemotactic cytokines (e.g., irg1l, il1, il1, ifn, il6, ccl20a, and tnf), which can result in inflammation, localized cellular damage, or death [64,65,66]. As a result, NPs may cause the over-reactivity of the immune system of aquatic species and potentially influence immune cell recruitment and responses to bacterial or viral infections (MoA immune system). 

These findings suggest that NPs can interact with critical components of the nervous system, such as enzymes involved in neurotransmitter metabolism. Their small size also allows them to accumulate in the brain, increasing their neurotoxicity. Behavioral alterations connected with NPs exposure may be linked to such changes, while more research is needed in this area. NPs, on the other hand, can cause inflammatory reactions through cytokine release, resulting in cellular damage and immune system changes. Finally, the neurotoxicity of NPs has been connected to alterations in the immune system, microbiota, and cellular metabolism [42], indicating a promising multidisciplinary area of investigation in the field of NP ecotoxicity.

### 3.6. The Effects of NPs Exposure on Animal Health and Fitness

The molecular and subcellular modifications generated by NPs in aquatic species can result in changes at the organ/tissue and individual/population levels [42,45] (MoA: organismal fitness). For example, metabolic shifts and changes in mitochondrial function by NPs (25–44 nm PS-NPs) [53,57,58] might alter energy balance and reduce growth potential, thus possibly influencing animal growth, development, reproduction, and success [67,68]. Inflammation and oxidative damage can also have an impact on animal growth and survival. According to a recent review [45], NPs-induced inflammation can affect these parameters by inducing the release of extracellular traps and neutrophil degranulation, in addition to tissue neutrophil infiltration, vacuolation, necrosis, lipidosis, and oxidative damage. These effects at higher biological levels strongly suggest that NPs are worldwide and serious hazards to environmental health. 

Changes in organismal fitness and health, as predicted, can be linked to a variety of subcellular or cellular processes. In summary, the antioxidant, metabolic, and immunological perturbances generated by NPs via hormonal, mitochondrial, or biochemical modifications (see Section 3.3, Section 3.4 and Section 3.5) are crucial to consider. Furthermore, as illustrated in Figure 2, the small size of NPs promotes their bioaccumulation in several tissues or organs, giving them the ability to potentially disrupt a wide range of physiological systems and processes, and ultimately impact organismal health and fitness. 

## 4. NPs as Carriers of Environmental Pollutants

The use of hydrophobic polymers in the manufacture of NPs and the associated high surface-area-to-volume or mass ratios imply that NPs may be effective transporters or carriers of a wide range of environmental pollutants [69,70]. This review will use the term “carrier” or “carrier effect” to refer to the ability of NPs to transport other chemicals and microorganisms on their surface. This term will not be used to discuss the possibility of NPs transporting other chemicals within their structure, such as stabilizers, plasticizers, and other compounds used in the synthesis of plastic polymers. The carrier effect has been examined by the scientific community because it has the potential to affect the health threat posed by NPs. Plastic particles can act as carriers of chemical compounds through adsorption or absorption. Adsorption occurs only at the solid–liquid interface and is most common at low pollutant concentrations due to strong hydrophobic, electrostatic, or non-covalent interactions between the plastic’s surface and the waterborne chemicals [71]. Absorption, on the other hand, is the process by which chemicals penetrate the plastic solid matrix as a result of partitioning [71]. A recently published review provides a complete description of the factors that drive the sorption of organic contaminants to plastic particles [71].

The analysis of MPs collected in field studies across the world shows that a wide range of pollutants and bacteria can sorb or bind to microscopic plastic particles. MPs collected in the Mediterranean Sea, for example, revealed a complex bacterial population, with *Polaribacter*, *Vibrio*, and *Loktanella* being the most prevalent taxa [72]. Biofilm formation in MPs transplanted to the Beilun River (China) for 30 days resulted in the enrichment of DNA sequences from microbial genes linked to antibiotic resistance, particularly when MPs were transplanted into urban areas [73]. Polycyclic aromatic hydrocarbons (PAHs), polychlorinated biphenyls (PCBs), metals, and pesticides were found adhering to the surfaces of MPs or bigger plastics collected on beaches along Brazil’s coastline [74,75,76]. There are no data on the carrier effect of environmental NPs, since their small size makes the recovery and identification of NPs in complex matrices difficult. However, much research has been conducted under laboratory conditions to study the role of NPs as potential carriers or vectors of pollutants and microbes, which will be discussed further in the following sections.

It is important to note that modeling studies have indicated that MPs may only play a small role in the transfer of contaminants to aquatic organisms. For example, according to laboratory studies, the plasticizers nonylphenol and bisphenol A are predicted to bioaccumulate in fish (cod) mostly through waterborne or gastrointestinal exposures. However, in certain cases, bisphenol A buildup from MPs ingestion may correspond to ambient levels of bisphenol A, as described in fish under severe conditions (i.e., very high plastic pollution and relatively high content of bisphenol A in the plastics) [77]. At the same time, as compared to other environmental matrices, such as water, air, dissolved organic carbon, organic colloids, black carbon, and biota, the content of organic contaminants sorbed to MPs is comparatively low. According to a recent review, MPs may concentrate organic contaminants from the water by seven orders of magnitude. However, the insignificant quantity of MPs in the oceans compared to water, biota, and organic carbon limits their significance as substantial vectors of aquatic contaminants [78]. Still, whether the same is true for NPs is debatable, given their greater surface-area-to-mass ratios, possibly higher ambient concentrations, and improved ability to cross biological barriers as compared with MPs.

In this section, we will look at how NPs interact with various types of environmental contaminants. It will first cover single pollutants, followed by brief subsections describing the recent and still limited findings on NPs and complex mixtures, microorganisms, and antibiotic-resistance genes (ARGs). Finally, we will review the current findings of the impacts of aging and weathering events on NPs and how they may affect the sorption or binding affinity of other pollutants, ultimately influencing the ecotoxicity of NPs under environmental conditions. Throughout this section of the review, we will look at how NP properties, such as size, charge, polymer composition, and coating, can affect pollutant sorption and the toxicity of pollutant-NP to aquatic species. The implications of environmental factors on NPs toxicity and sorption capacities will be discussed in Section 5.

### 4.1. Interactions of NPs with Environmental Pollutants and Resulting Toxicity to Aquatic Species

According to laboratory studies, the sorption of environmental contaminants to NPs is affected by particle properties, such as size and polymer type, as well as ambient conditions, such as natural organic matter (NOM). The sections that follow will go through the significance of these characteristics in terms of the carrier effect for specific contaminants and the resulting toxicity of pollutant-NPs to aquatic organisms. The majority of the research discussed below are laboratory studies, which can investigate the sorption of pollutants to NPs under controlled conditions. The data from this section are summarized in Table 1.

#### 4.1.1. Metals

Many investigations have shown that NPs can behave as metal transporters. Ionic silver (Ag^+^) and silver nanoparticles, for example, can both bind to the surface of NPs [92,95]. The sorption of Ag^+^ to PS-NPs occurs at varying rates and is affected by the particle size, composition, and concentration, as well as the presence of dissolved organic matter (DOM). When equivalent particle number concentrations are used, the surface area available for sorption in 600 nm PS-NPs is about two times that of 300 nm PS-NPs [92]. This increases the Ag^+^ sorption rate to NPs and the time required to reach a steady state [92]. The sorption of Ag^+^ to PE NPs (PE-NPs) was substantially lower than that of 300 nm and 600 nm PS-NPs, presumably due to the higher agglomeration rate and the resulting reduced surface area of PE-NPs [92]. In addition, at high concentrations, DOM will interact with the surface of NPs, affecting NPs aggregation and saturating the sorption of Ag^+^ to NPs [92]. According to the same study, the carrier effect of these NPs can increase the absorption and toxicity of Ag^+^ in daphnids due to the higher bioaccumulation rates and the ability of NPs to circumvent biological barriers. It is also suggested that, under realistic environmental conditions with high DOM concentrations, the carrier effect of NPs could be negligible [92]. 

Another study indicated that PS-NPs can also act as carriers for ionic silver derived from silver nanoparticles. The co-exposure of PS-NPs (10 nm) and silver nanoparticles in saltwater has been shown to promote Ag^+^ sorption to NPs (binding affinity 0.02 to 0.06 L/µg). This is probably a result of interactions of Ag^+^ with charged (carboxylated groups) or neutral areas of NPs [79]. PS-NPs seemed to enhance the permeability of the plasma membrane, as well as the bioaccumulation and toxicity of Ag^+^ in one of the examined marine microalgae species when co-exposed with silver nanoparticles [79]. In contrast, the results with the other examined species showed that NPs can compete with other nanoparticles for internal absorption, lowering the total bioaccumulation and toxicity of contaminants by the organisms [79].

According to a recent laboratory study, lead (Pb^2+^) bound to poly-dispersed NPs (150–450 nm) obtained from the breakdown of MPs collected on the shore of Baie Sainte-Marie in Guadeloupea (France) [94]. Pb^2+^ adsorbed rapidly and effectively to the NPs mixture, reaching a steady state in around 200 min. The findings indicated that sorption was related to chemical interactions between Pb^2+^ and the NPs surface, and was significantly dependent on pH values: higher pH enhanced the electrostatic interactions or covalent binding of the NPs surface with Pb^2+^ through the deprotonation of surface functional groups (e.g., R-COOH). Thus, the authors predicted that metal sorption to ambient NPs would increase owing to aging and weathering processes, which could result in the production of oxygenated surface functional groups, such as carboxylic acids, ketones, alcohols, hydroperoxides, and aldehydes. 

Metals do not always sorb to NPs, as originally expected. One study, for example, found that ionic copper (Cu^2+^) did not adsorb to COOH-functionalized PS-NPs (COOH-PS-NPs, 88 nm), which were employed as a surrogate for aged NPs [86]. As a consequence, the inclusion of COOH-PS-NPs did not affect the toxicity of Cu^2+^ to microalgae in the short or long term. According to the authors, this might be due to a poor affinity of Cu^2+^ for PS or due to the interaction of Cu^2+^ with freshwater medium components, preventing Cu^2+^ from interacting with the COOH-PS-NPs.

In summary, smaller particle sizes and lower polymer hydrophobicity improve the adsorption capacity/rate of plastics. This implies that small and less hydrophobic NPs may be stronger metal adsorbents than environmental MPs and have a higher ability to operate as free metal carriers when compared to other small plastics on a mass-to-mass basis. The presence of negatively charged groups on the surface of NPs may significantly increase the sorption of certain metals via electrostatic interactions, which is predicted in environmental NPs to be a result of aging and weathering activities. Furthermore, not only will the plastic-specific surface, porosity, and shape play important roles in metal adsorption to NPs, but so will water components, dissolved organic matter, and pH, as explored in greater depth in Section 5. As a result, the impact of the carrier effect on NP toxicity varies greatly depending on the metal, exposure medium inorganic and organic chemistry, plastic polymer, and surface functional groups.

#### 4.1.2. Polycyclic Aromatic Hydrocarbons (PAHs) and Polychlorinated Biphenyls (PCBs)

The sorption of PAHs to plastic particles is dependent on complicated interactions between the compounds’ exposed functional groups. For example, it has recently been demonstrated that, once in the water, PAHs such as benzo[a]pyrene (BaP) can take the form of self-aggregated nanoclusters, rather than free dissolved forms [96]. Insoluble BaP nanoclusters may stably bind to NPs (for example, 190 nm PS-NPs) and enter the cell via NP clathrin- and phagocytosis-mediated endocytosis. BaP nanoclusters can desorb from NPs and reach the cytosol or the endoplasmic reticulum during lysosomal transport and maturation, whereas NPs can bind to mitochondria or be expelled. NPs containing BaP nanoclusters resulted in substantial loss of mitochondrial membrane potential and triggered mitochondrial-dependent cellular apoptosis.

In mussels, the impact of the carrier effect of NPs on the transfer of NP-sorbed BaP was also studied [91]. Dietary exposure of mussels to BaP-spiked PS-NPs (500 nm) results in BaP transfer and bioaccumulation in the animals. BaP-containing PS-NPs were shown to be more hazardous than plain/virgin PS-NPs, affecting hemocyte lysosomal stability, the antioxidant system, the structural integrity of the digestive tubules, and the animal’s respiration rate. Thus, the carrier effect of NPs combined with the transfer of BaP to organisms greatly enhanced the toxicity of the PS-NPs via molecular to physiological changes.

There is relatively little known about the interactions between NPs and PCBs. For instance, PCBs interact with the aromatic structure of PS via hydrophobic and π–π-interactions at the large surface area of the PS-NPs (hundreds of times more than bulk PS) [84]. As a result of the higher surface-to-mass ratio, the sorption to PS-NPs (70 nm) can be 10^3^–10^6^ times greater than that to bulk PS (sorption coefficient 10^4^–10^9^ vs. 10^2.5^–10^3.1^ L/kg, respectively), and it can occur at a faster rate with planar (and more hazardous) PCBs than non-planar PCBs [84]. These findings show that PS-NPs can be effective PCB transporters with potential transfer to aquatic biotics, with the high lipid content of some tissues or organs facilitating PCB extraction from PS-NPs. Another study found that high concentrations (5 mg/L) of PS-NPs (100 nm) led to the scavenging of free PCB-18 from the water and PS-NPs would be the main driver of toxicity to daphnids [87]. On the other hand, low levels of PS-NPs will result in higher levels of free PCB-18 in the water, which will be the main inducers of toxicity to daphnids under this scenario [87]. 

Studies with MPs can also be used to improve the discussion of the potential interacting effects of NPs, PAHs, and PCBs on plastic behavior and toxicity. For example, studies with naphthalene (and its derivatives) as a model PAH showed that charged PAHs (e.g., -NH_2_, -OH, and -COOH) have greater molecular polarity and electrostatic surface potential, and reduced hydrophobicity (log Kow). As this is an important factor in PAH sorption to plastics, charged PAHs exhibited lower sorption capabilities (*Kd* between 6.0 and 8.4 L/g) towards 10 µm PS-MPs or COOH-PS-MPs than unmodified PAHs (*Kd* = 11.9 L/g) or uncharged PAHs (e.g., -CH_3_) (*Kd* = 11.6 L/g) [97]. Nitro-PAHs are another example of PAHs shown to interact with and modulate the toxicity of MPs. For instance, 9-nitroanthracene (9-NAnt) sorbs to 100–150 μm PE-, PP-, and PS-MPs (*Kd* = 34.0, 17.9, and 24.8 L/g, respectively). For PE-MPs, partitioning, hydrophobicity interactions, and van der Waals forces are at work, whereas sorption to PS-MPs or PP-MPs is explained by dissolution and hole-filling or molecular chemical adsorption, respectively [98]. Adult zebrafish exposed to 100–150 μm PE-MPs pre-sorbed with 9-NAnt accumulated NPAHs in the organisms, resulting in a delayed toxicity response with indications of neurotoxicity, altered energy metabolism, and intestinal dysbiosis [99].

In summary, the hydrophobicity of PAHs and PCBs facilitates interaction with NPs through various chemical interactions that rely on polymer type, surface chemistry, and PAH and PCB characteristics. In general, the addition of PAHs or PCBs to NPs enhanced the toxicity of the polymers to aquatic species, since the carrier effect can alter the routes of cellular absorption, intracellular transport, metabolism, and excretion of NP co-contaminants. This can affect the toxicity of both NPs and sorbed chemicals, resulting in the disruption of membrane structures, mitochondrial metabolism, and the digestive and immunological systems. 

#### 4.1.3. Pesticides, Biocides, and Antibiotics

Less is known about the interactions of pesticides, biocides, and antibiotics with NPs. Ciprofloxacin, for example, binds to COOH-PS-NPs (200 nm and 500 nm) via hydrogen bonding as well as electrostatic and hydrophobic interactions (adsorption capacities of 5.2 and 4.3 mg/g, respectively) [90]. The -COOH group of COOH-PS-NPs is protonated in acidic circumstances and negatively charged in alkaline situations. Thus, at the pH levels typical in aquatic settings (pH 6.5–8.5), its negative charge increases the electrostatic repulsion to ciprofloxacin, causing it to act as a zwitterion, rather than a cation (pH 6.1) or an anion (pH > 8.7). The adsorption affinity of 200 nm COOH-PS-NPs was greater than that of 500 nm COOH-PS-NPs, showing that smaller NPs may adsorb this antibiotic more effectively [90]. In the presence of COOH-PS-NPs, ciprofloxacin caused higher acute toxicity effects in the worm *Caenorhabditis elegans* than ciprofloxacin alone, reinforcing the role of the carrier effect in the toxicity of NPs [90]. 

In another study, tetracycline sorption on PS-NPs was also affected by the chemical structure of the NPs surfaces and the physicochemical properties of the water. For example, for plain and sulfonic-acid-modified (HSO_3_-) PS-NPs (100 nm), sorption occurs via unstable and weak contacts, while for NH_2_-PS-NPs, sorption occurs via a partition function [88] (for sorption coefficient comparisons, please see the original paper). Because this antibiotic has a neutral or negative charge, the lower zeta potential (more negative) of HSO_3_-PS-NPs and PS-NPs reduces tetracycline sorption as compared with the high (positive) value for NH_2_-PS-NPs. Higher salinities enhance the zeta potential of HSO_3_-PS-NPs and PS-NPs, as well as their hydrophobic interactions with tetracycline (salting-out effect), increasing tetracycline’s sorption to these two NPs. Tetracycline-containing SO_3_H-PS-NPs and PS-NPs were more hazardous to marine microalgae than their pristine counterparts owing to their greater zeta potential and lesser repulsion by the negative charge of the microalgae cellular membrane. Despite the positive zeta potential of NH_2_-PS-NPs, tetracycline sorption lowered the toxicity of this NP by increasing hydrophobicity and aggregation, as well as reducing NP absorption. 

Pesticides and fungicides can also bind to NPs, possibly modifying their toxicity via the carrier effect. The herbicide glyphosate binds to NH_2_-PS-NPs (203 nm) via the interaction of the reductive amino groups of the NP with the pesticide-oxidizing functional groups. This will result in substantial glyphosate elimination from the exposure medium (55–97 percent with 5 mg/L NH_2_-PS-NPs and 5–20 mg/L glyphosate). In this circumstance, NH_2_-PS-NPs protected microalgae against the toxicity caused by glyphosate. Simultaneously, the interaction between this pesticide and the NPs improved both the stability of the NH_2_-PS-NPs dispersion (increased hydrophilicity) and the NP adsorption to the microalgae [89]. Another study [82] found that the co-exposure of zebrafish larvae to PS-NPs (50 nm) and the fungicides ketoconazole or fluconazole exacerbated PS-NP toxicity by causing teratogenicity, cardiotoxicity, and oxidative stress, though it is unclear whether this was due to different toxicity mechanisms or increased bioaccumulation of the fungicides [82]. Thus, the presence of co-contaminants, such as pesticides or fungicides, might influence NP stability, as well as the absorption and bioaccumulation of both the NPs and co-contaminants, affecting the NPs’ toxicity.

Interestingly, NPs (50 nm PS-NPs) have also been demonstrated to block the function of the ATP-binding cassette transporters P-glycoprotein and multidrug resistance protein [81]. This is a major problem since it can impair the organism’s capacity to eliminate organic contaminants via efflux transporters. This idea was validated in the same investigation by exposing marine rotifers to the biocide triclosan following pre-exposure to PS-NPs. Triclosan toxicity was substantially enhanced in both acute and chronic exposures, as predicted, severely affecting animal survival, population growth, and reproduction [81]. 

In conclusion, the interactions of pesticides, biocides, and antibiotics with NPs were strongly influenced by pH, salinity, and the functional groups of the NPs, as these are all elements that affect electrostatic and hydrophobic interactions. Because NPs can function as pollutant transporters under favorable pH and salinity conditions, the sorption of these compounds to NPs enhanced the overall toxicity of the polymers. Furthermore, by interfering with the biotransformation and cellular excretion pathways, NPs can increase the persistence and toxicity of organic pollutants in complex environmental mixtures, including both types of toxicants.

#### 4.1.4. Other Compounds

Studies have indicated that a wide variety of organic compounds can sorb onto NPs or small MPs. According to research, a wide range of organic compounds can sorb to NPs or small MPs. For example, the synthetic phenolic antioxidant butylated hydroxyanisole, which is commonly used in the plastics industry, may rapidly bind to PS-NPs (65 nm). When zebrafish embryos are co-exposed to PS-NPs and butylated hydroxyanisole, the bioaccumulation of butylated hydroxyanisole and the developmental malformation rate rise, but the hatching, heart, and vertebrate calcification rates drop [83]. Furthermore, when co-exposed to butylated hydroxyanisole, PS-NPs were considerably more hazardous to energy metabolism, severely affecting lipid metabolism [83]. As a result, the combination of NPs and organic additives, as well as the carrier effect and the rapid sorption of organic pollutants to NPs, may produce more unfavorable health consequences in growing fish.

Flame retardants are another class of organic contaminants that can interact with and bind to plastics. For example, the flame retardant and plasticizer tris(1,3-dichloroisopropyl) phosphate (TDCIPP) achieved an adsorption equilibrium after 24 h, and adult zebrafish exposed to TDCIPP-containing NPs experienced the bioaccumulation of this chemical in the animals’ liver, gills, stomach, and gonads. Furthermore, as compared with waterborne TDCIPP exposure, this bioaccumulation resulted in TDCIPP paternal transmission and thyroid endocrine disturbance in the following generation (F1) [85]. The flame retardant triphenyl phosphate reduces locomotor activity and disrupts eye development in marine medaka embryos, although these effects are reduced in the presence of plain PS-NPs, COOH-PS-NPs, or aminated NH_2_-PS-NPs (1000 nm) [93]. This is probably due to triphenyl phosphate’s hydrophobicity, which facilitates its sorption to NPs while lowering the quantity of free triphenyl phosphate accessible in water to the organism [93].

The idea of small plastics functioning as one-way and two-way carriers of flame retardants in marine organisms has also been explored. The flame retardant polybrominated diphenyl ether (BDE-47), for example, has been demonstrated to increase the toxicity of PS-MPs (2 µm) in marine mussels via the carrier effect by reducing the animal feeding rate and causing oxidative stress and immunotoxicity [100]. Exposure to PE-MPs (10–45 m) pre-contaminated with polybrominated diphenyl ether (BDE-47) promotes the transfer of this flame retardant to marine amphipods [101]. However, due to the cleansing or reverse carrier effect, exposure to plain/virgin PE-MPs can reduce the quantity of BDE-47 bioaccumulated in species previously exposed to BDE-47 [101], suggesting that bioaccumulated small plastics can act as organic pollution scavengers and promote their excretion. As a result, it is important to note that the effects of plastics as two-way carriers or vectors of organic contaminants can play a significant role in the dynamics and toxicity of organic pollutants and plastic co-contaminants in aquatic ecosystems, a topic that has recently been studied with MPs, but is poorly understood or characterized with NPs.

#### 4.1.5. Complex Environmental Chemical Mixtures

There is relatively little information available on the interactions and toxicity of NPs in the presence of complex mixtures, indicating a significant research gap in the field. To strengthen the discussion of this subject, we integrated data on NPs with research on small MPs (at the low micrometer range) to better understand how the toxicity of tiny plastics, such as NPs, might be affected in complex pollutant mixtures.

Water-accommodated fractions (WAFs) of petroleum are complex mixes of organic contaminants that include a high concentration of PAHs and have been widely investigated in ecotoxicology. Recent research utilized a WAF made from crude oil, which resulted in a combination of at least 13 detected PAHs, the most prevalent of which were naphthalenes and fluorenes [80]. The co-exposure of marine rotifers to WAF and PS-NPs (50 nm) enhanced PS-NP toxicity, resulting in decreased population growth and molecular changes associated with altered reproduction and energy reallocation toward adaptability. Extensive transcriptomics analyses show that PS-NP exposure is linked to altered cell cycle regulation and energy balance. WAF exposure activated antioxidant responses, kidney proliferation, and fatty acid metabolism. The combination of PS-NPs and WAF, on the other hand, was highly linked to mitochondrial dysfunction (protein and nucleotide metabolism, as well as oxidative stress induction), but antioxidant responses, stress, and heart toxicity were also relevant toxicity pathways. Thus, it is evident that NPs can not only enhance the toxicity of complex PAH combinations but also modulate toxicity processes, potentially resulting in synergistic effects with negative ecological implications.

Two studies conducted by our group used zebrafish as a model organism to explore the impact of NPs on the toxicity of a complex combination of PAHs. A sediment extract produced from a contaminated location in the Elizabeth River, Virginia (USA), was used in these experiments as a complex real-world environmental mixture, including 36 identified PAHs. This combination is extremely hazardous to growing fish, affecting animal survival and circulatory system development [58]. While plain PS-NPs (44 nm) were not harmful to growing fish, they did reduce the bioaccumulation and developmental toxicity of this PAH combination in a co-exposure scenario [58]. This was most likely induced by the sorption of free PAHs to the PS-NPs, which resulted in aggregation and reduced NP and co-contaminant uptake by the organisms. Despite this protective impact, PS-NPs were highly toxic to mitochondria, reducing ATP and NADH synthesis [58]. In other research, embryonic zebrafish were exposed to either plain PS-NPs (44 nm) or PS-NPs spiked with PAHs from the same PAH mixture mentioned above [57]. The impact of the carrier effect of NPs resulted in the transmission and bioaccumulation of PAHs to the organisms in this situation. In particular, it changed the tissue biodistribution of PAHs as compared with free PAH exposure, moving PAHs to organs and possibly organelles with greater PS-NP bioaccumulation, such as the brain and mitochondria. Furthermore, the sorption of PAHs to PS-NPs exacerbated the disturbance of energy metabolism and the mitochondrial toxicity of NPs [57]. 

A recent study with Norway lobsters (*Nephrops norvegicus*) investigated the sorption of tiny MPs (6 µm and 500–600 µm) to a combination of ten PCB congeners (PCBs 28, 52, 101, 118, 138, 153, 180, CB29, CN112, and CB140) [102]. The results indicated that the sorption of these chemicals to the surface of PE- or PS-MPs (155 mg) successfully eliminated (>73 percent removal) the PCBs included in the mixture (**∑**10 PCBs = 1.5 g or 13.5 g). Because of the greater surface area (0.95 m^2^/g for 6 µm PS-MPs versus 0.01 m^2^/g for 500–600 µm PS-MPs), this sorption was more effective with the smallest MP tested (6 µm PS-MP and 97 percent removal of PCBs from the solution). Despite the passage of PCBs through the digestive system and their bioaccumulation in the lipid-rich tail tissue, exposing lobsters to small MPs pre-loaded with PCBs did not affect the animal’s condition factor, nutritional status, or tail lipid content.

Surprisingly, the carrier effect of plastics implies that they may play a role in the reverse movement of contaminants, such as PCBs. According to a recent study, small and virgin MPs (4 µm) aided in the depuration of persistent organic pollutants, such as PCBs, in daphnids [103]. Animals pre-contaminated with a combination of PCBs (PCB 18, 40, 128, and 209) depurated PCB 209 quicker following exposure to small MPs. In another study, rainbow trout (*Oncorhynchus mykiss*) previously given food contaminated with PCBs (PCB 18, 40, 128, and 209) and later exposed to medium-size PE-MPs (212–250 µm) did not, however, present the same reverse carrier effect on PCB [104]. These findings emphasize the need for gathering information on how plastic physicochemical properties might change the effects of MPs and NPs on the bioaccumulation and toxicity of organic contaminants to various aquatic species.

Finally, a recent study looked at the ability of PP-MPs to bind to a complex mixture of metal ions [105]. The results showed greater adsorption of the transition metals Cr, Co, Cu, Zn, and Fe, as well as virtually all of the rare earth elements examined. According to the authors, rare earth metals are a group of elements with similar chemical characteristics that may co-precipitate as carbonate complexes and deposit on MPs’ surfaces. The sorption of rare earth metals to MPs can also be accelerated in surfactant-coated MPs due to increased ionic interactions between metals and the Ca or Mg in the surfactant.

Small polymers, particularly NPs, may interact differently at the individual level in the presence of complex combinations of environmental contaminants. The adsorption affinity of many environmental toxicants may vary widely, potentially boosting the sorption of certain pollutants at the expense of others. As a result, the toxicity of freshly formed pollutant-NPs will be influenced by the chemical composition of the pollutants coupled with the NPs. Furthermore, the reverse carrier effect will influence the toxicity of complex mixtures by changing the profiles of bioaccumulated substances in organisms. This can have a significant impact on animal health, especially for persistent pollutants that are more difficult to metabolize and excrete.

### 4.2. Interactions of NPs with Microorganisms and Antibiotic-Resistance Genes

Microbial biofilms on plastic waste are another example of how plastic particles are affected by their surrounding environment. This is a major problem. since plastics may function as transporters of microorganisms and diseases (REF), as well as promote the spread of antibiotic-resistance genes (ARGs) [106]. The sorption of organic materials as a soft layer on the plastic surface improves the carrier effect by increasing the hydrophobicity and contact angle with water [107]. Because dissolved organic matter sorbs with varied affinities to different plastic polymeric surfaces and aging/weathering conditions, this sorption is selective [107]. The presence of an organic layer on the plastic surface not only aids bacterial colonization but also changes the microbial makeup of the plastic (i.e., selective bacterial enrichment) in comparison to the ambient or inoculating population [107]. Because there is relatively little knowledge regarding this process with NPs and the subsequent toxicity of biofilm-containing NPs, we will share data with MPs to further address this issue.

A recent review summarized the role of MPs as ARG carriers [106]. The majority of the available data in this study indicated that wastewater treatment facilities, landfill leachates, aquaculture systems, urban water, and aquatic habitats are important sources of ARG enrichment in MPs. Water treatment plants should be given special consideration, since they might enhance the presence of contaminants, such as antibiotics and heavy metals, throughout the treatment process. This can also lead to an increase in the abundance of ARGs on MPs, particularly in sludge. Plastics with varying polymers, additives, or co-contaminants may have varying ARG compositions, since these variables can promote the selective enrichment of certain bacteria and ARGs. Other variables influencing ARG-plastic enrichment include ambient biota, pH, nutrients, salinity, temperature, and hydrodynamics. As a result, ARG-plastics can be adsorbed or absorbed by bacterial communities and aquatic plants, and they can also be consumed by aquatic animals. This can result in the horizontal and vertical gene transfer of ARGs in the former and microbial dysbiosis and digestive system inflammation in the latter. For further details on these processes and the effects of ARG-MPs in the aquatic environment, readers should refer to the review referenced above [106].

Once in the aquatic environment, plastics are quickly colonized and engulfed by NOM and a biofilm of bacteria known as the plastisphere [108]. Field studies with in situ transplanted MPs films indicate that the biofilms that constitute the plastisphere can be recognized as metabolic hotspots [109]. They will differ in composition and concentration from the surrounding surface waters, and, as recently reviewed, they can potentially harbor hazardous microorganisms and pathogens (for example, the dinoflagellates *Ostreopsis* sp. and *Coolia* sp., the bacteria or cyanobacteria *Tenacibaculum* sp., *Phormidium* sp., and *Leptolyngbya* sp., and the ciliate *Halofolliculina* spp.) [108]. This can result in the spread of diseases through aquatic ecosystems, as well as influence the impacts of small plastics on aquatic animals. 

Some studies have indicated that plastics coated with a biofilm of microbial communities can be more detrimental to the aquatic environment. For example, under laboratory circumstances, coating 45 µm polymethyl methacrylate MPs (PMMA-MPs) with *Escherichia coli* enhances MP uptake and cellular respiration in marine oysters. The identical but virgin PMMA-MPs caused no physiological changes in the organisms [110]. PS-MPs (45 µm) coated with bacterial biofilm also accumulated at a faster rate in exposed sea urchins than virgin PS-MPs, causing greater egestion rates, decreased immune cell counts, changes in the antioxidant system, and increased generation of reactive oxygen and nitrogen species [111]. In another study under laboratory conditions, the inclusion of a real-world environmental biofilm enhanced detritivorous amphipods’ shredding rate of high-density polyethylene (HDPE) plastics (1 cm); however, it was unclear if the shredded particles were rejected or eaten by the organisms [112]. This was most likely due to the biofilm attracting these detritivorous amphipods as a feeding cue, as stated by the authors.

The existence of a biofilm on the surface of plastics can also change the composition of contaminants that are sorbed to the polymers. According to a field study using transplanted PE-MPs in situ, increased biofilm biomass in MPs resulted in a reduction in the amount of low- and medium-molecular-weight PAHs, but not PCBs, sorbed to PE-fibers [113]. This is most likely because some of the bacteria concentrated in the PE-fibers were known to break down PAHs [113]. Furthermore, it is important to highlight that, based on laboratory studies, the interaction of NPs with bacteria might result in NP aggregation and deposition in aquatic settings. Cyanobacteria, for example, may form aggregates with 100 nm PS-NPs with the aid of extracellular polymeric compounds, particularly in seawater. This causes increased NP density and sinking, altering NP transport and destiny in aquatic settings [114]. 

A recent field study with shrimps collected from natural ponds in China proposed that the bacterial populations in small plastics may alter the host’s gut microbiome, thereby indirectly influencing plastic toxicity [115]. Furthermore, because environmental plastics may accumulate in the digestive tracts of species at various trophic levels [28,29,30], they have the potential to influence the gut microbiota [116]. Alterations to innate immune responses can also alter the gut microbiota by favoring the dominance of more resistant bacterial groups and disrupting the physiological functions of the organism [116]. Furthermore, the plastic additives that leach from the plastics can have negative consequences on the gut–microbiome axis. When adult zebrafish were co-exposed in the laboratory to titanium dioxide nanoparticles (240 nm), there was evidence that bisphenol A caused the dysbiosis of the gut microbiota. Surprisingly, this alteration occurred as a result of antagonistic interactions at low BPA concentrations and synergistic interactions at high BPA concentrations [117]. In another study in which adult zebrafish were fed 3 ppm of the plastic additive diethyl-hexyl phthalate under laboratory conditions, helper T cells were significantly upregulated [118]. These findings suggest that small plastics and their additives can influence the microbiome−gut−immune axis. However, research on this issue has just recently begun, and more research is needed to better understand the interactions between NPs, the gut microbiota, and the organism’s physiological homeostasis.

To the best of our knowledge, the data regarding NPs and the topics discussed above are very limited, if not non-existent, revealing a huge gap in understanding regarding the toxicity mediated by the carrier effect and biofilms with small polymers. 

### 4.3. Effects of Aging and Weathering of Plastic Particles on the Carrier Effect

Plastic particle aging or weathering caused by abiotic (e.g., abrasion, UV radiation, and sunlight) or biotic (e.g., bacterial degradation) factors can affect the sorption rate of pollutants to plastics due to physicochemical changes on the particle’s surface. According to laboratory studies, aging can increase the particle surface area and roughness and break polymer molecular bonds in small plastics [119]. This can result in the release of ROS and the oxidation of functional groups, thereby increasing the adsorption of pollutants, such as antibiotics [119,120] and metals [121], to plastics. Photoaged PS-MPs (125–250 µm), on the other hand, exhibit a reduced sorption coefficient against more hydrophobic molecules, such as apolar aliphatics, monopolar aliphatics, bipolar aliphatics, non-polar aromatics, monopolar aromatics, and bipolar aromatics [122]. Plastic aging or weathering in marine environments has the intriguing effect of decreasing biofilm development and attachment while increasing the synthesis of extracellular matrix polymeric components by marine bacteria, which improves cell adherence to roughened surfaces [123]. 

Data on the aging and weathering of MNPs are quite sparse, especially for NPs, representing a significant scientific gap in our understanding of the carrier effect in real-world settings. Recent data indicate that UV light alters the surface chemical structure of PS-NPs (100 nm), increasing the abundance of oxygen-containing functional groups [124]. As expected, this results in increased hydrophilicity at the same time as decreased sorption to organic molecules, such as humic acid, lysozyme, and alginate. As a general rule, we can expect that UV will increase the surface area and decrease the zeta potential of small plastics, also enhancing their adsorption capacity for some organic compounds by shifting the predominant π–π interactions to electrostatic and hydrogen-bonding interactions [125]. 

It is crucial to remember that NPs have a large surface-area-to-mass ratio that might be affected by aging and weathering processes. Furthermore, NPs can be produced in the environment by the gradual decomposition of MPs and bigger plastic particles. Thus, we propose that NPs’ high surface area and lengthy durations of exposure to abiotic or biotic variables may imply a greater vulnerability to aging or weathering processes, eventually influencing NPs’ toxicity and the carrier effect of these small polymers.

## 5. Effects of Other Environmental Stressors on NPs’ Toxicity and Interactions with other Environmental Pollutants

A variety of variables, including concentration, exposure length, particle condition, shape, polymer type, and other environmental conditions, influence the toxicity of MNPs to aquatic life. Many recent reviews examined the impact of these variables in modifying MNPs’ toxicity to aquatic species in terms of their development, population growth, physiological stress, oxidative stress, energy metabolism, and immune and endocrine systems. However, less emphasis has been given to how environmental factors contribute to MNP toxicity in aquatic settings. To further discuss the effects of environmental conditions on plastic toxicities, several environmental variables, including temperature, salinity, pH, natural organic matter, and food availability, were included in the current review to investigate the multi-stressor consequences on plastic toxicity, and are discussed more below. The data discussing NPs and MPs in this section are summarized in Table 2.

### 5.1. Temperature

The research described in this section has shown that temperature influences plastic absorption and toxicity, stressing the need for considering temperature when assessing the toxicity of NPs to aquatic organisms. Temperature’s effects on COOH-PS-NPs’ (500 nm) toxicity were studied with the water flea, *Daphnia magna*, at 18 and 24 °C [126]. COOH-PS-NPs had a greater influence on immunity at higher temperatures, as shown by the increased number of immune cells (hemocytes). Another study [127] found that raising water temperature enhanced the susceptibility to small MPs toxicity in two temperate Cladoceran species, *D. magna* and *Daphnia pulex*, as well as a smaller tropical species, *Ceriodaphnia dubia*. The authors investigated the effects of both primary plastic microspheres (1–5 µm, polymer unknown) and secondary PE-MPs (1–10 µm). In *D. magna* and *D. pulex*, acute toxicity sensitivity to both forms of MPs increased with temperature, whereas *C. dubai* did not. An additional study investigated the effects of water temperature on the toxicity of PE-MPs (1–5 μm) in *D. magna* [128] but found no link between rising water temperatures (from 20 °C to 25 °C) and changes in population fitness caused by MPs. Recent data suggest that the toxicity of small plastics under warmer conditions can be amplified when paired with additional stressors. For example, the toxicity of PS-MPs (5–100 µm) to *D. magna* was synergistically affected when paired with a higher water temperature and ammonium, exerting the greatest impact on survival rates via diminishing filtering capacity [129]. 

In addition to the water flea experiments, the effects of temperature on MNP toxicity in fish have been examined. For example, common goby fish (*Pomatoschistus microps*) were used to study the toxicity of cefalexin in connection to the presence of PE-MPs (1–5 µm) and increasing water temperature [130]. In animals exposed to PE-MPs, increased water temperatures increased the death rates and decreased predatory performance. Another study [131] in this line discovered that high temperatures enhanced PE-MPs absorption (70–88 μm), but inhibited the deleterious effect of MPs on the predatory performance in Discus fish *(Symphysodon aequifasciatus*). PE-MPs prevented (completely or partially) the increase in amylase and lactate dehydrogenase activity or decrease in citrate synthase activity caused by higher temperatures. Overall, it appears that higher temperatures will have a detrimental influence on the digestive capacities and raise the anaerobic metabolic demand of the organisms. The presence of MPs at higher temperatures can amplify such effects on the digestive system and shift the metabolic demand towards aerobic respiration. As a result, these studies demonstrate the significance of evaluating the effects of temperature in understanding plastic toxicity in aquatic life.

### 5.2. Salinity and pH

Salinity plays an important role in organisms that inhabit the marine environment and influences the toxicity of other environmental pollutants. However, there is little evidence concerning the influence of salinity on NPs’ toxicity. It has been reported that high salinity, along with naturally occurring microorganisms in marine areas, will accelerate the breakdown of plastics, increasing their availability to aquatic organisms. We reviewed papers that explored the impacts of salinity on MNPs’ toxicity and overall suggested that it is an important element in the stability of small plastic particles in aqueous solutions. There are two basic concepts on how salinity influences the fate and toxicity of small plastics [141]: higher salinity either (1) neutralizes the surface charges of plastics by compressing their electric double layer and decreasing electrostatic interactions in the sorption of plastics to other contaminants, or (2) induces the salting-out effect of other chemicals and partitioning chemicals between water and plastics. The following studies showed the effects of salinity on NPs’ aggregation, toxicity, and interaction with other contaminants. To improve the discussion, MP-related papers were also examined.

Several investigations have assessed the role of salinity in altering the physicochemical characteristics and aggregation of MNPs. One study [132] analyzed the influence of salinity on the aggregation behavior and toxicity of PS-NPs (100 nm) with different functional groups: plain PS-NPs, COOH-PS-NPs, negatively charged NH_2_-PS-NPs, and positively charged NH_2_-PS-NPs. Using a salinity gradient ranging from 0 to 35‰, the authors detected that high salinity resulted in significant aggregation and accumulation of NPs in the sediment, thereby increasing the potential risk of these NPs to benthic species. Furthermore, the authors found that, when the salinity rose, the zeta potential values of both positively and negatively charged NPs dropped. According to the interaction energy profiles of Derjaguin–Landau–Verwey–Overbeek, low salinity induces greater repulsion between NPs and a stable state. However, when the salinity increases, the electrostatic double layer compresses, resulting in a drop in repulsive forces and a decrease in the net repulsive energy barrier between the NPs. This process results in aggregation and a significant influence of salinity on the fate, stability, transport, and toxicity of NPs, as well as their ecological consequences under diverse environmental conditions. In another study, the effects of salinity on the agglomeration rate were investigated in NPs with various core compositions (dyed PS-NPs with red or blue) and surface chemistry (plain PMMA-NPs or COOH-PMMA-NPs) [133]. The authors found that the effects of salinity varied with the surface chemistry of NPs, suggesting that COOH-PMMA-NPs agglomerated regardless of the salinity, whereas plain PMMA-NPs required salinity values of 1 g/L or higher. No difference was detected in the agglomeration pattern of red and blue PS-NPs. 

Several additional studies suggested that salinity also plays a key role in the sorption of pollutants to NPs. For example, low to medium salinity values (5 to 15‰) increased the sorption of the antibiotic ciprofloxacin to PS-NPs (40 nm), though high salinity (25 and 35‰) decreased it. This occurs as increasing salt concentrations cause the formation of NPs clusters due to the compression of the electric double layer at the adsorbent surface of the NPs, increasing the electrostatic interaction with ciprofloxacin. However, at extremely high salinity, the adsorption capacity may be reduced as NaCl competes for the interaction with NPs [134]. Bisphenol A adsorption to NPs, on the other hand, is mainly governed by hydrophobic interactions with a limited increase in adsorption capacity at higher salinities due to the salting-out effect [134]. This finding suggests that NPs have varying affinities for pollutants, which are influenced by salinity. Furthermore, increased sorption of PCBs to MNPs was reported [84]. The authors studied the sorption of 17 PCB congeners to PE-MPs (10−180 μm), PS-NPs (70 nm), multi-walled carbon nanotubes (MWCNT), and fullerene (C60). Salinity decreased sorption for MWCNT, but increased sorption for the PE-MPs and PS-NPs. The sorption capacity of metals to plastics has also been shown to alter with salinity variations. For example, the sorption of Cr to plastics increases at higher salinities while it decreases for Co, Cd, and Ni [142]. Overall, the effect of salinity on NPs sorption behavior is determined by the plastic particle and pollutant characteristics. Increased salinity, in general, decreases the solubility of non-polar and polar organic contaminants in water and neutralizes the surface charges of plastics by compressing the electric double layers. As a result, the sorption of non-polar and polar organic contaminants to NPs will be increased [143]. 

Compared to salinity, less research has been undertaken to study the impact of pH on small plastics. A previously reported study [144] also investigated the effects of pH on the sorption capabilities of sulfamethoxazole on PE-, PS-, PET-, PVC-, and PP-MPs (100–150 µm) and discovered that the sorption capacity decreased as pH increased. Another study found that increasing the pH of the water reduced the time required to establish equilibrium between perfluoroalkyl compound sorption and HDPE-MPs (3–16 µm), PS-MPs (10 µm), and PS-COOH-MPs (10 µm). The results showed that increasing the pH of the water containing salts reduced the propensity of PFASs to sorb onto plastics [135]. Similarly, depending on the polymer and chemical tested, the sorption behavior of two perfluorochemicals on PE-MPs (150 µm), PS-MPs (250 µm), and PVC-MPs (230 µm) microplastics [145] can either increase or not change with reduced pH. At low pH, the surfaces of PE-MP and OS-MP particles can be protonated, resulting in a positive surface that can sorb anionic perfluorochemical molecules. These data imply that pH has a major impact on the sorption capacity of NPs and other small plastics to other pollutants and potentially modulates the toxicity of the plastic particles.

### 5.3. Natural Organic Matter (NOM)

DOM (the dissolved portion of NOM) can interact with plastics through complex mechanisms, but few studies have looked at the relationship between MNPs and DOM in aquatic ecosystems. DOM has been discovered as being attracted to plastic surfaces by a variety of adsorption processes, including electrostatic attraction, hydrophobic interactions, and ligand exchange [146]. Adsorption to the surfaces of natural colloids and artificial nanoparticles in aquatic environments may impact their fate and transit by altering their surface characteristics and aggregation behavior, hence influencing the sinking rates [147,148]. Humic substances (HSs), in particular, may attach to MNPs [143,149] to generate an eco-corona (EC) or a protein corona (PC) [136]. MNPs are hydrophobic molecules that bind to proteins produced by the metabolism of aquatic organisms, such as extracellular polymeric substances (EPS) and/or the exoproteome, leading to the formation of an EC. Adsorption to endogenous proteins, on the other hand, produces PC. Additionally, as already discussed (Section 4.1), it is known that DOM/NOM may influence the sorption of chemicals to MNPs. These mechanisms, in combination, impact the bioavailability, transport, degradation, and toxicity of plastic particles.

Evidence for NP toxicity in the presence of organic molecules was shown to be conflicting. The relationship between three HSs, namely fulvic acid (FA), NOM, and humic acid (HA), and the corona formation and toxicity of PS-NPs (about 110 nm), was studied in *D. magna* [136]. PS-NPs’ acute toxicity was reduced by HSs, which also altered the expression of genes involved in endocrine function, detoxification, and oxidative stress. More specifically, NOM and HA decreased gene expression while FA increased it, which might be related to HA’s lower adsorption and FA’s larger adsorption, which increased EC and PC synthesis. Similarly, the toxicity of several PS-NPs to *D. magna* was reduced by the presence of HA, probably by decreasing the aggregation of the NPs and reducing the entanglement and body burden on the organisms [132].

The effect of DOM on the stability, transport, and dispersion of nanoparticles in a water column has been widely investigated, and it may vary based on particle characteristics, such as size and chemical composition. Particle charge, for example, has a considerable influence on DOM sorption. Negative charges inhibit polysaccharide and biopolymer aggregation on particle surfaces due to charge stabilization [150], but positive charges may increase it [132]. Additionally, the colloidal stability of nanoparticles in an aquatic environment in relation to several variables has been investigated. The ionic strength, capping agent, electrolyte valence, and pH govern the colloidal stability of gold nanoparticles [151], but NOM may alter nanoparticle stability by outweighing these characteristics, interacting with Ca^2+^ [59], and coating particle surfaces [152]. Overall, these data highlight the need for considering the effects of DOM when determining the fate, transport, and toxicity of MNPs in aquatic settings. 

### 5.4. Food Availability

Food availability, in addition to the abiotic parameters outlined above, is critical in determining MNPs’ toxicity to an organism. According to our knowledge, there are no studies examining this problem with NPs; hence, we utilized MPs as examples of another form of small polymers, whose toxicity is known to be modified by food availability. On the influence of food supply on plastic toxicity, two contradicting occurrences are explained. 

There is evidence that most aquatic species, such as copepods, can discriminate between food particles and counterfeit particles [153,154], preventing them from eating artificial plastic particles in the presence of an abundant food supply. In one investigation, *D. magna* were exposed to PS-MPs (2 μm) in the presence or not of algae (*Chlorella vulgaris*), and their growth, mortality, and reproduction were investigated [137]. The results showed that, in the presence of natural food, *D. magna* consumed fewer MPs, alleviating the MP risk in the context of an abundant food supply. Similarly, daphnids that were exposed to PS-MPs (15 μm) [138] also found that MP ingestion was reduced in the presence of algae. 

In contrast, another study found that *D. magna* could not differentiate between MPs and food particles in the medium [155]. The impact of food availability on primary MPs (1–5 μm, polymer unknown) and secondary MPs (PE-MPs, 2.6 µm) uptake was studied using *D. magna*. MPs exhibited a strong detrimental influence on algae intake (particularly secondary PE-MPs), reducing the growth rate and producing other negative consequences on reproduction, regardless of algal abundance [139]. Another study investigated the effect of PE-MPs (20 μm) on arctic copepods, including *Calanus finmarchicus*, *C. glacialis*, and *C. hyperboreus* [140], as a factor of food availability. The study used various combinations of MPs and diets and found no effect on food availability, as PE-MP intake increased with increasing concentrations of MPs, independent of food content.

Despite contradicting findings regarding the influence of food availability on MNPs’ toxicities, the majority of research suggests that the toxicity of small plastics decreases under laboratory conditions with adequate food supply.

## 6. Perspectives for Future Research in the Field

**Figure 3:**
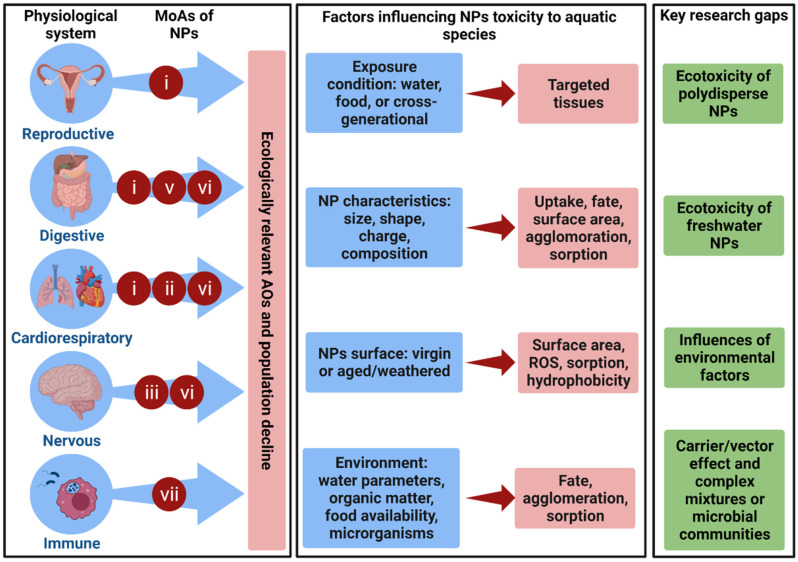
**Summary of the effects of nanoplastics (NPs) on aquatic species, their toxicity in response to the interactions with organismal and environmental parameters, and main research gaps in the field.** (**Left panel**): NPs may accumulate in tissues related to key physiological systems, triggering a cascade of molecular, biochemical, cellular, and physiological processes that can explain many of the observed or predicted ecologically relevant adverse outcomes of NPs. This chain of events may be broken down into seven modes of action (MoAs, please see Section 3.2 for further clarification): (i) organismal fitness, (ii) circulatory and respiratory systems, (iii) behavioral, sensory, and neuromuscular function, (iv) alimentary and excretory systems, (v) microbiome, (vi) metabolism, and (vii) immune system [42]. (**Center panel**): differences in NPs’ properties and exposure pathways, whether caused by environmental conditions or not, might result in altered behavior, fate, uptake, sorption to pollutants or microorganisms, hydrophobicity, or the formation of reactive oxygen species (ROS). (**Right panel**): examples of research gaps that may indicate future directions to consider in the field of NPs ecotoxicity (please see Section 4 for clarification). Organs are shown as corresponding to mammalian species for purposes of visualization.

As shown in this review, a growing body of studies has focused on the aquatic toxicity of nanometer-scale plastic particles or fragments (NPs), suggesting multiple modes of action and interactions with organismal and environmental parameters (Figure 3). Despite such remarkable improvement, there are significant issues in the field of NPs ecotoxicity that remain unresolved or understudied.

There are still several challenges in the field of NPs toxicity to aquatic species. For example, a recent study characterized research topics underrepresented in the field of NPs [156]: only 9% of the studies focus on the separation and analysis of NPs, while 8% characterized the generation of NPs through the fragmentation of larger plastics or the fate, behavior, and transport of NPs. Other major environmental concerns, such as the aging and weathering processes, as well as the impact of the carrier effect of NPs, are similarly underrepresented (9%). Because the emphasis of this study is on the ecotoxicity of NPs to aquatic species, we would like to highlight subtopics of major environmental relevance that have received little attention or investigation in this area. 

The first concerns the ecotoxicity of polydisperse or complex NP mixtures. MNPs are present in the environment in a broad range of particle sizes, from nanometers to millimeters (5 mm), with a wide range of shapes (e.g., fragments, films, spheres, foams, and fibers), colors, weathered conditions, and polymer types [157]. The adverse effects on aquatic organisms will be affected by each of these parameters and their combinations, considerably increasing the complexity of MNPs ecotoxicity assessment. As a result, one major gap in NPs ecotoxicity research is the current use of single and monodispersed NPs, which oversimplifies the plastic pollution problem. The utilization of complicated and, if feasible, real-world mixes of MNPs will be a critical step in advancing research on this subject. 

In addition, the relative lack of research on the ecotoxicity of NPs in freshwater ecosystems is a second source of concern. The majority of plastics research has been conducted in the marine environment, although inland rivers and other freshwater habitats are also recipients of plastics contamination and are widely recognized as significant contributors to marine plastic pollution [158]. Because freshwater bodies have water chemistry and ambient circumstances distinct from those of marine waters, MNPs may behave differently in these systems. Thus, it is crucial to broaden our research to include the identification, characterization, behavior, and toxicity of NPs in freshwater environments.

The next subtopic we wish to emphasize as a critical need in NP research is how environmental factors or stressors play a role in NP toxicity to aquatic species. As discussed in this review, temperature, salinity, NOM, and food availability can all alter NPs’ toxicity. Animal physiology may be adversely affected if water temperatures rise over the ideal setpoint, thereby potentially affecting the toxicity threshold of NPs. Salinity, pH, and organic matter, on the other hand, will impact the physicochemical characteristics of NPs, possibly leading to significant changes in NPs’ behavior, fate, transport, bioaccumulation, and interactions with other contaminants. Finally, it is vital to note that certain aquatic animals can distinguish between food particles and MPs, whilst others cannot. It is unknown if this is also true for small polymers, such as NPs; thus, further studies are necessary to investigate the potential impacts of NPs on feeding selectivity and different animal nutrition. These aspects must be recognized and investigated in laboratory studies on the toxicity of NPs to aquatic species. This will allow us to better replicate or represent a diverse variety of aquatic ecosystems and their seasonal or temporal fluctuations, enhancing our understanding of the potential effects of NPs in complex and real-world settings. 

The final subtopic we would like to highlight is the importance of considering the impacts of the carrier effect of NPs in the context of complex pollutant mixtures or microbial communities. Much research has been undertaken to investigate how pollutants interact with MNPs, although the majority of these studies employed only one kind of pollutant and one type of plastic particle. As we discussed in Section 2, NPs can greatly concentrate contaminants found in the water column or sediment, but not all pollutants interact with NPs with the same affinity. Thus, in a real-world scenario of complex mixtures, NPs may cause the differential enrichment of pollutants available in the environment, which will vary depending on the physicochemical characteristics of each NP particle. In addition, pollutants already adsorbed to NPs will impact the resulting interaction of NPs with additional environmental pollutants. Other key issues in this field include (i) how multiple environmental factors, such as temperature, pH, salinity, and organic matter, will affect these interactions; (ii) how such complex and variable enrichment of co-contaminants on NPs’ surfaces will impact NPs’ toxicity; and (iii) how weathering might impact the physicochemical characteristics of not only the NPs but also their adsorbed pollutants. All the questions outlined above also apply to the interactions of NPs with microbe communities, rather than environmental pollutants.

Plastic pollution is, without a doubt, a huge environmental concern, and these are only a few of the pressing needs and challenges in the dynamic and diverse subject of plastic’s ecotoxicity. We are now dealing with unprecedented quantities of plastic waste in our food, water, and environment, and it is critical that the scientific community discovers solutions to many of the problems posed in this area. We expect that this summary of current research on the toxicity of NPs to aquatic species will contribute to the discussion of the impacts of small plastics, specifically NPs, on our aquatic environments by accounting for their interactions with water parameters, contaminants, and environmental conditions.

## Figures and Tables

**Table 1:** Summary of the interactive effects of nanoplastics (NPs) and other environmental pollutants.

NP Type * and Size ^#^	Additional Environmental Pollutant	Effects Detected	References
PS-NP (10 nm)	Ag^+^ from silver nanoparticles	Ag^+^ reacts with neutral or charged PS-NPs. Depending on the species, sorption of Ag^+^ promotes or lowers bioaccumulation and toxicity to microalgae.	[79]
PS-NP (44 nm)	Complex PAH mixture (sediment extract)	Co-exposure decreases PAH bioaccumulation and developmental toxicity in developing fish through sorption to NPs and lower water PHAs levels. NPs impair mitochondrial energy production.	[58]
PS-NP (44 nm)	Complex PAH mixture (sediment extract)	PAH-spiked NPs transfer PAHs to the brain and the yolk sac, and cause greater mitochondrial toxicity in developing fish.	[57]
PS-NP (50 nm)	Complex PAH mixture (crude oil WAF)	Co-exposures increase PS toxicity, impairing rotifer reproduction, mitochondrial function, energy metabolism, and population growth.	[80]
PS-NP (50 nm)	Triclosan	NPs block efflux proteins, increasing triclosan’s toxicity and bioaccumulation.	[81]
PS-NP (50 nm)	Ketoconazole or fluconazole	Co-exposure enhances the developmental and cardiovascular toxicity to developing fish.	[82]
PS-NP (65 nm)	Butylated hydroxyanisole	Co-exposure increases bioaccumulation of this synthetic antioxidant in developing fish and impairs fish growth and energy metabolism.	[83]
PS-NP (70 nm)	Various PCB congeners	PCB interactions with PS aromatic structure occur via hydrophobic and π–π interactions.	[84]
PS-NP (70 nm)	TDCIPP	TDCIPP binds to NPs and accumulates in fish gonads and digestive organs. NPs and TDCIPP are transferred to offspring and cause endocrine dysregulation.	[85]
COOH-PS-NP (88 nm)	Cu^2+^	Cu^2+^ does not bind to COOH-PS-NPs or alter toxicity to microalgae.	[86]
PS-NP (100 nm)	PCB-18	Sorption of PCB-18 increases NPs toxicity to daphnids. Low doses of NPs reduce the toxicity of PCB-18 by decreasing free PCB-18 levels in the water.	[87]
PS-NP, HSO_3_-PS-NP, and NH_2_-PS-NP (100 nm)	Tetracycline	Tetracycline binds to plain or HSO_3_-PS-NPs through weak interactions or to NH_2_-PS-NPs by partition function. Tetracycline (neutral or negatively charged) has restricted sorption to negatively charged NPs. Higher salinity increases NPs zeta potential and sorption of tetracycline. NPs containing tetracycline were more toxic to microalgae.	[88]
NH_2_-PS-NP (23 nm)	Glyphosate	Glyphosate binds to NH_2_-NPs by the interaction of oxidizing and reducing functional groups. NH_2_-NPs favor the stability and uptake of glyphosate by microalgae.	[89]
COOH-PS-NP (200 and 500 nm)	Ciprofloxacin	Smaller NPs have a greater adsorption affinity towards ciprofloxacin. Sorption to NPs increases the toxicity to nematodes.	[90]
PS-NP (500 nm)	BaP	Exposure to BaP-spiked PS-NPs promotes BaP bioaccumulation in mussels, affecting the immune, digestive, and antioxidant systems.	[91]
PS-NP and PE-NP (300 and 600 nm)	Ag^+^	Greater surface areas for sorption of Ag^+^ in 300 nm NPs. Higher PE-NPs agglomeration reduced surface area and Ag^+^ sorption compared to PS-NPs. NOM decreases the sorption of Ag^+^ to NPs. Sorption of Ag^+^ increases NPs toxicity to daphnids.	[92]
PS-NP (1000 nm)	Triphenyl phosphate	NPs can reduce the toxicity of triphenyl phosphate to fish embryos by decreasing free triphenyl phosphate levels in water	[93]
Various (140–450 nm)	Pb^2+^	Higher pH values favor Pb^2+^ sorption to NPs through electrostatic interaction or covalent binding via NPs functional group deprotonation.	[94]

* Surface group functionalization is represented by COOH, NH_2_, or HSO_3_. ^#^ The nominal value from a commercial source or the value measured using electron microscopy or dynamic zeta potential (hydrodynamic diameter). To keep it concise, only data addressing NPs are included, while research reporting MPs is not. Abbreviations: PAHs stands for polycyclic aromatic hydrocarbons; PCBs stands for polychlorinated biphenyls; TDCIPP stands for tris(1,3-dichloroisopropyl) phosphate; and WAF stands for water accommodated fraction.

**Table 2:** Summary of the interactive effects of nanoplastics (NPs) and other small plastics (<100 μm) with environmental parameters or stressors.

Environmental Factors	Plastic Type * and Size ^#^	Main Effect	References
Temperature	COOH-PS-NPs (500 nm)	Temperature increases the impact of genotypic immunological responses of daphnids to plastics.	[126]
Temperature	MPs (1–5 µm, polymer not described) and secondary PE-MPs (1–10 µm)	Rising temperature increases acute sensitivity to MPs in daphnids, but not in ceriodaphnids.	[127]
Temperature	PE-MPs (1–5 μm)	Daphnid MP-driven population fitness is unaffected by high temperatures.	[128]
Temperature	PS-MPs (5–100 µm)	Co-exposure to elevated temperatures and ammonia increases the impacts of MPs on daphnid survival and feeding rate.	[129]
Temperature	PE-MPs (1–5 µm)	Higher water temperatures increase MP-exposed fish mortality and decrease predatory performance.	[130]
Temperature	PE-MPs (70–88 μm)	Temperature rise enhances MP bioaccumulation but does not affect fish survival or predatory performance.	[131]
Salinity	PS-NPs COOH-PS-NPs, and NH_2_-PS-NPs (100 nm)	High salinity causes NP aggregation and sedimentation.	[132]
Salinity	PMMA-NPs, COOH-PPMA-NPs, and blue and red PS-NPs (55 to 62 nm)	Effects of salinity on NP aggregation depend on NP composition and surface chemistry.	[133]
Salinity	PS-NPs (40 nm)	The sorption capacity of NPs to ciprofloxacin and bisphenol A increases as salinity increases. However, it is reduced at extreme salinities.	[134]
Salinity	PE-MPs (10−180 μm) and PS-NPs (70 nm)	Higher salinities increase the sorption of PCBs to PE-MPs and PS-NPs.	[84]
pH	HDPE-MPs (3–16 µm), PS-MPs (10 µm), and PS-COOH-MPs (10 µm)	Higher pH values enhance the sorption of perfluoroalkyl compounds to MPs.	[135]
Natural organic matter	PS-NPs (110 nm)	Natural organic matter, humic acid, and fulvic acid reduce the acute toxicity of PS-NPs to daphnids. Natural organic matter and humic acid mitigate the expression of genes related to detoxification, oxidative stress, and endocrine activity.	[136]
Natural organic matter	PS-NPs (50–300 nm)	Decreases aggregation of NPs and toxicity to daphnids.	[132]
Food availability	PS-MPs (2 μm)	Daphnids select natural food over MPs with abundant food supply.	[137]
Food availability	PS-MPs (15 μm)	Reduces ingestion of MPs by daphnids at higher algal levels.	[138]
Food availability	PE-MPs (2.6 µm), MP (1–5 μm, polymer not described)	Food availability has no impact on MP ingestion in daphnids	[139]
Food availability	PE-MP (20 µm)	Food availability does not affect food ingestion in copepods	[140]

* Surface group functionalization is represented by COOH, NH_2_, or HSO_3_. ^#^ The nominal value from a commercial source, or the value measured using electron microscopy or dynamic zeta potential (hydrodynamic diameter). Due to the limited number of studies with NPs, data addressing MPs are also included but narrowed to small MPs up to 100 µm. Abbreviations: PCBs stands for polychlorinated biphenyls; PFOS stands for perfluoro octane sulfonate; and FOSA stands for perfluorooctanesulfonamide.

## Data Availability

Not applicable.
